# The effect of cottonseed oil on lipids/lipoproteins: a systematic review and plasma cholesterol predictive equations estimations

**DOI:** 10.1093/nutrit/nuad109

**Published:** 2023-09-11

**Authors:** Tricia L Hart, Kristina S Petersen, Penny M Kris-Etherton

**Affiliations:** Department of Nutritional Sciences, Penn State University, University Park, PA, USA; Department of Nutritional Sciences, Penn State University, University Park, PA, USA; Department of Nutritional Sciences, Penn State University, University Park, PA, USA

**Keywords:** cottonseed oil, lipids, Katan equation, lipid metabolism, lipoproteins

## Abstract

**Context:**

Cottonseed oil (CSO) is higher in polyunsaturated fatty acids (PUFA) and saturated fatty acids (SFAs) than many liquid plant oils.

**Objectives:**

To conduct a systematic review of randomized controlled trials (RCTs) examining effects of CSO on markers of lipid metabolism and evaluate lipid and lipoprotein effects of incorporating CSO into a healthy dietary pattern using regression equations.

**Data Sources:**

A systematic search was conducted for RCTs comparing CSO with a non-CSO comparator in any population.

**Data Analyses:**

The Katan regression equation was used to predict lipid/lipoprotein changes when incorporating CSO into a US-style healthy eating pattern at 25 to 100% of the total oil allowance (ie, 27 g/2000 kcal) compared with average American intake (NHANES 2017 to 2020 pre-COVID pandemic).

**Results:**

In total, 3 eligible publications (n = 2 trials), with 58 participants that provided 44% and 30% of total energy as CSO, were included. Fasting low-density lipoprotein cholesterol (LDL-C; ≈ –7.7 mg/dL) and triglycerides (≈ –7.5 mg/dL) were lower after 5 days of a CSO-enriched diet vs olive oil (OO). In a 56-day trial, CSO lowered total cholesterol (TC; ≈ –14.8 mg/dL), LDL-C (≈ –14.0 mg/dL), and non–high-density lipoprotein cholesterol (≈ –14.2 mg/dL) vs OO. Postprandially, angiopoietin-like protein-3, -4, and -8 concentrations decreased with CSO and increased with OO intake. Compared with average American intake, a healthy eating pattern with 27 g of CSO was estimated to lower TC (–8.1 mg/dL) and LDL-C (–7.3 mg/dL) levels, with minimal reduction in high-density lipoprotein cholesterol (–1.1 mg/dL). Compared with the healthy eating pattern, incorporating 27 g of CSO was predicted to increase TC and LDL-C levels by 2.4 mg/dL.

**Conclusion:**

Limited high-quality research suggests CSO may improve lipid/lipoprotein levels compared with OO. Cholesterol predictive equations suggest CSO can be incorporated into a healthy dietary pattern without significantly affecting lipids/lipoproteins.

## INTRODUCTION

Cardiovascular disease (CVD) is the leading cause of death globally, accounting for about 17.9 million deaths in 2019.^1^ In the United States, approximately 50% of adults have CVD.^2^ Diet is a key modifiable risk factor for CVD. In 2016, approximately 52% of premature deaths (∼9.1 million) due to CVD were related to dietary risk factors.^3^^,^^4^ Dietary fatty acids significantly influence CVD risk, with saturated fat acids (SFAs) increasing, and unsaturated fatty acids (both polyunsaturated fat [PUFA] and monounsaturated fat [MUFA]) decreasing levels of low-density lipoprotein cholesterol (LDL-C), a major CVD risk factor.^5^ Cottonseed oil (CSO) is becoming more common in the food supply, resulting from higher demand, more efficient production,^6^ and use in parenteral nutrition formulas.^7^ CSO is higher in SFA (26%) and PUFA (52%) than other commonly consumed oils (eg, soybean oil, mixed vegetable oils) including high MUFA oils (eg, canola oil, olive oil). In this review, we explore the effects of CSO on markers of lipid metabolism, including lipids and lipoproteins (hereafter, lipid/lipoprotein), body weight, and angiopoietin-like proteins (ANGPTLs), and hence, CVD risk.

Management of dyslipidemia is a primary target for atherosclerotic CVD risk reduction.^8^ A key component of dietary recommendations for dyslipidemia is replacement of SFA with unsaturated fats.^8^ However, substitution of SFA with PUFA results in greater lipid/lipoprotein lowering than substituting with MUFA. PUFAs regulate lipids/lipoproteins through many mechanisms.^9^ Recent evidence suggests high-PUFA diets may modulate ANGPTLs to improve lipid/lipoprotein levels.^10^ ANGPTLs are generating much interest as regulators of lipid metabolism that offer potential as therapeutic targets for management of dyslipidemia.^11^^,^^12^ ANGPTL-3, -4, and -8 regulate the clearance of circulating triglycerides via inhibition of lipoprotein lipase.^11^ Importantly, ANGPTL-related regulation of lipoprotein lipase appears to influence LDL-C and apolipoprotein B levels.^13^ ANGPTLs may also have a role in body fat accumulation^14^ and obesity-related impairment of lipid/lipoprotein metabolism.^15^

The fatty acid composition of CSO, as well as the amount of phytosterols (sterols and stanols 44 mg/15 mL),^16^ and possibly of dihydrosterculic acid (∼0.3%),^17^ would be expected to improve lipid/lipoprotein levels; however, limited research has been conducted with CSO and, to our knowledge, there is no summary of this evidence. Thus, we conducted a systematic review of randomized controlled trials (RCTs) that examined the effect of CSO on lipid/lipoprotein metabolism. In addition, to better understand the potential effect of CSO on lipids/lipoproteins, we used a blood-cholesterol predictive equation to estimate how inclusion of CSO in healthy dietary patterns would affect lipids/lipoproteins.

## METHODS

A systematic review was conducted in accordance with the Cochrane Collaboration Handbook for Systematic Reviews of Interventions^18^ to investigate the quantity and quality of research evaluating the effect of CSO on markers of lipid metabolism, including lipids/lipoproteins, body weight, and ANGPTLs. This review was not registered, and the protocol was not posted before completion. This review was funded by Cotton Incorporated. The funders had no role in the methodology implemented or interpretation of data.

### Eligibility criteria

RCTs published in English comparing CSO with a non-CSO comparator in any population were included. Studies that did not include the outcome of interest or had concomitant interventions that did not enable the independent effect of CSO to be estimated were excluded. Studies that included the intervention and outcome of interest but did not meet all inclusion criteria (eg, not randomized) were included in a qualitative synthesis to document the quantity of research on the topic. See Table 1 for inclusion and exclusion criteria.

**Table 1:** PICOS criteria for inclusion of studies

	Inclusion criteria	Exclusion criteria
Population	Humans, all ages, any health status	Rodent models, in vitro models
Intervention	Intake of cottonseed oil	Concurrent interventions that did not enable the independent effect of CSO to be estimated (e.g., weight loss)
Comparison	Diet or condition not containing cottonseed oil	Comparator contained cottonseed oil in a different amount than the intervention
Outcomes	Lipids and lipoproteins, postprandial angiopoietin-like proteins, body weight	Any outcome not specified as an inclusion criterion; no comparison of the outcome(s) between the intervention and comparator condition at follow-up
Study design or publication type	Randomized controlled trials	Nonrandomized trials, observational studies, reviews, conference proceedings or abstracts

### Search strategy

Searches were conducted on May 15, 2022, of the following databases: PubMed, Clinicaltrials.gov, Cochrane Central, Cumulative Index to Nursing and Allied Health Literature (CINAHL), and CAB with no date restrictions imposed and publications limited to those published in English. The Supporting Information online includes the search terms used. All search results were uploaded to Rayyan-Intelligent Systematic Review for eligibility assessment.^19^ Duplicates were removed prior to title and abstract screening. Two authors independently conducted the title and abstract screening (T.L.H. and K.S.P.). All full text articles were reviewed independently by the same 2 authors. Disagreements were resolved by discussion among the authors.

### Data extraction

Data were extracted from all studies that remained after title and abstract screening into a spreadsheet by 1 author (T.L.H.) and reviewed by 2 other authors (K.S.P. and P.M.K.-E.). The data extracted included the study design, intervention type, length of intervention, participant characteristics (ie, age range, health conditions), sample size, percentage of male participants, intervention including the amount of CSO, comparator condition, outcome measures (ie, lipids/lipoproteins, ANGPTL, and body weight), and results. Lipids/lipoproteins, cholesterol esters/ and triglyceride data in both the fasted and fed states were extracted. Differences between the CSO condition and the control condition are reported as mean difference.

### Risk of bias

Risk of bias was assessed via the Cochrane Risk of Bias tool by 1 author (T.L.H.) and reviewed by a second author (K.S.P.).^18^ Risk was identified as low, medium, high, or unclear for all included studies.

### Predicted effects of fatty acids on lipid/lipoprotein profile

The Katan equation^20^ was used to predict lipid/lipoprotein differences when including CSO in a recommended healthy eating pattern compared with a healthy eating pattern without CSO and with average American intake. The US-style healthy eating pattern recommended by the 2020–2025 Dietary Guidelines for Americans was used as the healthy recommended eating pattern.^21^ The nutrient composition of this pattern, including the oil component (27 g/2000 kcal), was based on the Dietary Guidelines Advisory Committee (DGAC) 2020 report.^22^ The fatty acid composition of the oil component was based on the DGAC modeled oil composite (ie, unhydrogenated soy oil [53%], canola oil [22%], corn oil [10%], olive oil [OO] (4%), CSO [4%], and peanut oil [1%]).^22^^,^^23^ This oil composite represents the anticipated nutrient profile of oils consumed in the United States based on food supply data.^22^ What We Eat in America 2017 to pre-COVID pandemic 2020 data and the 2020 DGAC report were used to estimate average American intake.^22^^,^^23^ The fatty acid composition of CSO was obtained from the US Department of Agriculture Food Central Database.^24^ Expected lipid/lipoprotein changes with a healthy dietary pattern with 25%, 50%, 75%, and 100% of the oil allowance as CSO was compared with average American intake. In addition, we compared a healthy dietary pattern with a healthy dietary pattern containing CSO as 25%, 50%, 75%, and 100% of the oil allowance.

## RESULTS

### Systematic search

**Figure 1:**
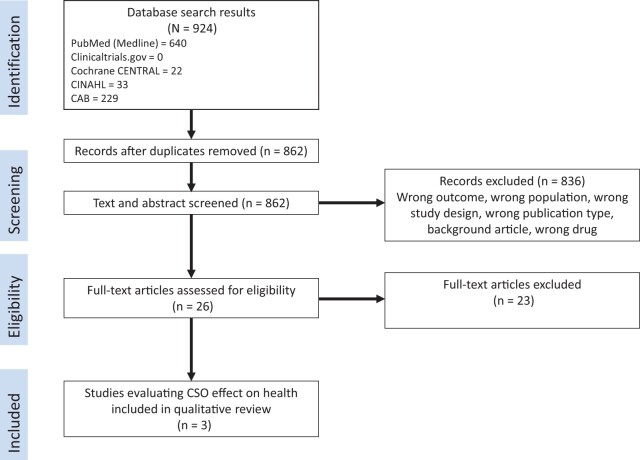
Flow diagram of the literature search process. *Abbreviations:* CINAHL, Cumulative Index to Nursing and Allied Health Literature; CSO, cottonseed oil.

Initial database searches returned 924 results; after duplicates were removed, 862 articles remained. Title and abstract screening resulted in the removal of 836 articles prior to full-text review. Of the 26 full-text articles reviewed, only 3 publications (n = 2 trials) met the criteria for inclusion (Figure 1). Reasons for article exclusion are reported in Figure 1.

### Eligible studies

The 3 eligible publications (Table 2)^10^^,^^25^^,^^26^ reported results from 2 trials (durations of 5 days and 56 days) and had a total of 58 participants (n = 15 and n = 43 in each study, respectively). The other publications were excluded from further analysis because of high risk of bias. Participants consumed 44% of total energy or 30% of total energy as CSO, respectively. The studies included were funded by the National Cottonseed Products Association and Cotton Incorporated, with disclaimers that sponsors had no role in the design, implementation, data analysis, or interpretation of results. Included studies had low risk of bias and were published between 2018 and 2022.

**Table 2:** Eligible full-text articles identified by the systematic search strategy^a^

Reference	Design	Duration, d	Participant characteristics	Sample size, no.	Intervention	Control	Outcome measure	Results
Polley et al, 2018^25^	Randomized crossover controlled feeding	5	Adults, 18–45 yo, healthy	15 (100% male)	44% of total energy as CSO	44% of total energy as OO	Fasting and postprandial lipids	LDL-C: ↓ CSO vs OO (∼ –7.5 mg/dL) TG: ↓ CSO vs OO (∼ –7.7 mg/dL) No other between-diet differences in fasting or postprandial outcomes
Kaviani et al, 2021^10^	Randomized, crossover, controlled feeding	5	Adults, 18–45 yo, healthy	15 (100% male)	44% of total energy as CSO	44% of total energy as OO	Fasting and postprandial ANGPTL-3, -4, and -8	ANGPTL-3: Change pre- to post-diet visit was different between CSO and OO at 120-min after breakfast and lunch (both *P* < 0.05) ANGPTL-4: Change pre- to post-diet visit was different between CSO and OO at 120 and 240 min after breakfast and 120 min after lunch (all *P* < 0.05) ANGPTL-8: Change pre- to post-diet visit was different between CSO and OO 240 min after lunch (*P* = 0.03) No other between-diet differences in fasting or postprandial outcomes
Prater et al, 2022^26^	Randomized parallel partial feeding	56	Adults, 43–63 yo, hypercholesterolemia	43 (37% male)	30% of total energy as CSO	30% of total energy as OO	Blood lipids	TC: ↓ CSO vs OO (∼ –14.82 mg/dL) LDL-C: ↓ CSO vs OO (∼ –13.98 mg/dL) Non–HDL-C: ↓ CSO vs OO (∼ –14.2 mg/dL) apoB: ↓ CSO vs OO (∼ –8.7 mg/dL) No other between-diet differences in fasting or postprandial outcomes

a Risk of bias for all 3 studies was low.

*Abbreviations:* ANGPTL, angiopoietin-like protein; apoB, apolipoprotein B; CSO, cottonseed oil; HDL-C, high-density lipoprotein cholesterol; LDL-C, low-density lipoprotein cholesterol; OO, olive oil; TC, total cholesterol; TG, triglyceride; yo, years old.

### Fasting and postprandial lipids

Both studies measured fasting blood lipids/lipoproteins. In the 5-day trial,^25^ fasting LDL-C and TG levels were significantly reduced following CSO intake compared with the control condition (OO). In the longer trial (56 days), there were treatment by visit interactions for TC (*P* = 0.027), LDL-C (*P* = 0.04), and non–HDL-C (*P* = 0.04) that were driven by a decrease in the CSO group and no change in the OO group.^26^ High-fat liquid meal challenges were conducted in both trials. In the 5-day trial, a liquid meal challenge comprising 35% of daily energy (1% milk, chocolate-flavored whey protein, chocolate syrup, and added OO or CSO oil) was provided at breakfast and 240 minutes later for lunch at both baseline and end-point visits. There was a significant time effect (*P* < 0.001), visit effect (*P* < 0.001), and treatment by visit interaction (*P* < 0.001), but no differences between groups.^25^ In the 56-day trial, 2 liquid meals (each containing 35% of daily energy) that participants consumed at breakfast and 240 minutes later contained 69.5% of energy from fat and were high in saturated fat (46.9% of energy) provided by unsalted butter, red palm oil, coconut oil, soy lecithin granules, and powdered-chocolate drink mix. These postprandial trials were completed at both baseline and end-point visits. This trial observed a treatment by visit interaction for postprandial TG levels. TG levels were increased with OO at all time points from 30 to 300 minutes (*P* < 0.05), with no change following the CSO intervention.^26^ There was no effect of oil type on body weight.^26^

### Fasting and postprandial angiopoietin-like proteins 3, 4, and 8

Only the 5-day trial analyzed ANGPTLs. There were no significant differences in fasting ANGPTL-3 concentrations between CSO and OO. There was a significant time by treatment interaction for ANGPTL-3 postprandially (at 120 minutes after breakfast and lunch) with decreases in the CSO group and increases in OO group (*P* < 0.05 for both).^10^

Like ANGPTL-3, fasting concentrations of ANGPTL-4 did not differ between groups. Postprandially, ANGPTL-4 levels decreased in the CSO group at 120 minutes after breakfast and lunch and 240 minutes after breakfast and increased in the OO group at these time points (*P* < 0.05 for all).^10^ There was a significant treatment effect postprandially at 240 minutes after lunch with decreases in ANGPTL-8 concentrations for the CSO group and increases in the OO group (*P* < 0.05).^10^

### Excluded studies

Twenty-three of 26 studies implemented an intervention that included CSO (Figure 1) but were excluded because other inclusion criteria were not met. Reasons for exclusion based on specific eligibility criteria were as follows: study design (n = 18), population (n = 1), comparator (n = 1), outcome (n = 1), or publication type (n = 2). A qualitative summary of the excluded studies is presented in Table S1 in the Supporting Information online. Because of the high risk of bias, these studies were excluded from further evaluation.

### Katan equation calculations

The fatty acid compositions of the dietary patterns used for the Katan equation calculations are presented in Table 3.^21^^,^^23^ Compared with average American intake, a healthy eating pattern with 25% to 100% of the oil allowance as CSO would be expected to lower TC (–10.1 to –8.1 mg/dL), LDL-C (–8.9 to –7.3 mg/dL), and HDL-C (–2.4 to –1.1 mg/dL), and increase TG (+8.9 to +2.4 mg/dL). Intake of a DGAC-modeled healthy eating pattern including CSO at any level studied (ie, 25%, 50%, 75% of the oil component) was predicted to change lipids/lipoproteins by less than 2.5 mg/dL compared with the DGAC-modeled healthy eating pattern (Table 4).

**Table 3:** The fatty acid profiles of the dietary patterns used to examine predicted lipid and lipoprotein changes with incorporation of cottonseed oil

Fat composition (% of total energy)	AAI^23^	HD^22^	HD + 100% CSO	HD + 75% CSO	HD + 50% CSO	HD + 25% CSO
Total fat	36.03	28.95	29.05	29.05	29.05	29.05
Saturated fat	12.43	5.87	7.49	7.09	6.68	6.28
MUFA	11.47	10.27	8.03	8.59	9.15	9.71
PUFA	8.20	10.46	10.99	10.86	10.72	10.59

*Abbreviations:* AAI, average American intake; CSO, cottonseed oil; HD, healthy US diet; MUFA, monounsaturated fatty acid; PUFA, polyunsaturated fatty acid.

**Table 4:** Katan plasma-cholesterol predictive equation estimated changes in lipids and lipoproteins when incorporating cottonseed oil into a US-style healthy eating pattern, compared with average American intake and the pattern recommended by the 2020–2025 Dietary Guidelines for Americans^21^

Lipid or lipoprotein (mg/dL)	AAI to HD with 100% CSO	AAI to HD with 75% CSO	AAI to HD with 50% CSO	AAI to HD with 25% CSO	HD to HD with 100% CSO	HD to HD with 75% CSO	HD to HD with 50% CSO	HD to HD with 25% CSO
TC	−8.1	−8.9	−9.7	−10.1	2.4	1.8	1.2	0.6
LDL-C	−7.3	−7.7	−8.1	−8.9	2.4	1.8	1.2	0.6
HDL-C	−1.1	−2.3	−2.4	−2.4	0.1	0	0	0
TG	2.4	8.7	8.8	8.9	−0.5	−0.4	−0.2	−0.1
Total/HDL-C	−0.091	−0.026	−0.038	−0.049	0.046	0.035	0.023	0.012
apoA	−2	−4	−3.9	−3.9	−0.1	−0.1	−0.1	0
apoB	−3	−2.1	−2.3	−2.6	1.1	0.8	0.6	0.3

*Abbreviations:* AAI, average American intake; apoA, apolipoprotein A; apoB, apolipoprotein B; CSO, cottonseed oil; HD, healthy US-style diet; HDL-C, high-density lipoprotein cholesterol; LDL-C, low-density lipoprotein cholesterol; TC, total cholesterol; TG, triglyceride.

## DISCUSSION

In this systematic review, only 2 trials (n = 58 participants) evaluating the effect of CSO vs OO on cardiovascular risk were identified that met our inclusion criteria. The findings from these studies suggest that CSO may improve lipid/lipoprotein concentrations and differentially affect ANGPTL-3, -4, and -8 compared with OO. Fasting TG and LDL-C concentrations were ∼8.8% lower after 5 days of consuming a diet enriched with CSO compared with OO, with no other fasting or postprandial differences observed between the oils.^25^ In the 56-day trial, the CSO-enriched diet, compared with the OO-enriched diet, elicited significant reductions from baseline in TC (–7.38% change vs –0.96% change), LDL-C (–12.16% change vs –3.54% change), non–HDL-C (–11.9% change vs –3.93% change), and apolipoprotein B (–10.5% change vs –2.79% change).^26^ Postprandially, ANGPTL-3, -4, and -8 levels decreased with CSO from baseline and increased with OO intake. Despite the limited evidence, both the 5- and 56-day study results suggest that intake of CSO may improve lipids/lipoproteins compared to OO.

The results of the reviewed studies align with those of previous studies that have evaluated the effect of dietary fatty acids on lipids/lipoproteins. Previous research has demonstrated a greater cholesterol-lowering effect of high PUFA oils, such as corn oil^27^ and sunflower oil,^28^ compared with high MUFA OO. A recent meta-analysis of 12 studies (n = 1089 participants) evaluated the effects of omega-6–rich oils (ie, corn, sunflower, and primrose oils) compared with OO. Omega-6–rich oils resulted in greater TC reductions compared with OO (weighted mean difference, –9.9; 95%CI, –17, –2.75), but no difference between groups for LDL-C (weighted mean difference, 5.23 (95%CI, –0.2, 10.5).^29^ SFA increases HDL-C levels,^30^ which, along with the increase in LDL-C, contributes to an increase in TC. On the basis of this evidence, the high proportion of omega-6 PUFAs from CSO would be expected to attenuate the increased LDL-C response to the SFA.

The Katan equation results suggest that CSO can be included in a healthy dietary pattern, with modest effects (< 2.5 mg/dL) on the lipid/lipoprotein profile even when 100% of the recommended 27 g of oils is replaced with CSO, which is improbable. When consuming a variety of oils, CSO can be incorporated (∼25%–50% of all oil) with even smaller effects on LDL-C and TC (<1.3 mg/dL). Compared with average American intake, a healthy dietary pattern containing CSO is predicted to reduce TC and LDL-C levels when up to 100% of the oil component was CSO. Although CSO is higher in SFA than other nontropical plant oils, the healthy diet with CSO was still low in SFA (∼6% kcal) and aligns with the dietary guidelines for Americans recommendations to consume a diet with <10% of energy from SFA.^21–23^

The regression equation estimates were smaller than those observed in the 5- and 56-day trials. The Katan regression equation was created using data from well-controlled clinical trials estimate the effect of fatty acid intake on serum lipid/lipoprotein levels.^20^ Results obtained using the Katan equation closely align with changes reported in multiple studies.^31^^,^^32^ The greater lipid/lipoprotein effects observed with CSO in the 2 trials identified in our systematic review suggest that the phenolic compounds in CSO may confer further cholesterol-lowering benefits beyond the fatty acid profile alone.^33^ Phenolics like plant sterols and stanols, which are found in plant oils, grains, fruits, nuts, and vegetables, reduce LDL-C levels by decreasing intestinal cholesterol absorption and increasing LDL-C receptor expression,^33^^,^^34^ which results in lower circulating LDL-C levels. Intake of 2 g/d plant sterols and stanols decrease LDL-C levels by up to 10%, with comparable benefits with up to ∼3 g/d.^33^^,^^34^ In the studies discussed, CSO would have contributed ∼3.2 g/2000 kcal phytosterols, which could explain the LDL-C reductions observed (∼9% and ∼12%).

Other possible explanations for the larger-than-expected effect sizes in the 2 CSO trials relate to the composition of the rest of the experimental diet. The 56-day trial was a partial feeding study; therefore, the actual composition, including the fatty acid content, remains unknown because of reliance on self-reported dietary data.^26^^,^^35^ The duration of the second study was only 5 days,^25^ which is likely insufficient time for lipid/lipoprotein responses to stabilize. Also, the diets provided may have been an improvement over the participants’ habitual diet. Furthermore, the 56-day trial only included individuals with hypercholesterolemia.^26^ It is well documented that people with higher baseline lipid/lipoprotein levels have greater reductions in response to a cholesterol-lowering dietary intervention.^36^

Another possible contributor to lipoprotein metabolism is ANGPTLs. The effect of dietary fat composition on ANGPTL-3, -4, and -8 is largely unknown. We investigated how the fatty acid composition of a diet containing CSO may affect ANGPTL. Postprandial effects of the high-fat meals were small, and the physiological implications remain unclear. Chylomicrons were not evaluated, and triglycerides were not different between groups, supporting the need for further research to clarify the effect of diet on ANGPTLs and the metabolic implications.

There are several limitations to this systematic review. First, only 3 publications from 2 trials were eligible for inclusion. Interestingly, several studies of CSO were conducted in 1950–1960.^37–39^ Several studies evaluated the effects of CSO provided as tube-feeding formulas on serum cholesterol and triglyceride levels.^40^^,^^41^ In the past decade, there has been renewed interest in CSO because of greater availability^42^ and possibly because of its fatty acid profile. The included studies had relatively small sample sizes and short durations. Another important limitation is that both trials used OO as the comparator, which is a high-MUFA oil. This is not representative of usual oil intake in the United States, where soybean oil, high in PUFA, is the most commonly consumed oil.^22^^,^^23^ Although these results provide evidence that CSO may offer benefits over high-MUFA oils, more research is needed to examine the lipid/lipoprotein effects of CSO compared with other higher PUFA oils that are lower in SFA. Limitations of the review process include lack of preregistration and limiting the search to English publications, which may have narrowed search results.

## CONCLUSION

This systematic review of CSO suggests beneficial effects on lipid/lipoprotein metabolism compared with that of OO beyond what is expected from fatty acid profile alone.^25^^,^^26^ Predictive equations support that CSO can be incorporated into a healthy dietary pattern, especially in small amounts, without substantially altering lipid/lipoprotein concentrations. Well-designed future studies should evaluate CSO’s effects on lipid/lipoprotein metabolism compared with oils that are commonly consumed in the United States and further investigation of the effect on ANGPTLs is warranted.

## Supplementary Material

nuad109_Supplementary_Data

## Supporting Information

The following Supporting Information is available through the online version of this article at the publisher’s website.

*Table S1* **Qualitative summary of studies identified by the systematic search strategy and excluded from full-text review**

*Table S2* **Search terms by search engine with filters in parentheses**
