# Supplementary material for: The effect of cottonseed oil on lipids/lipoproteins: a systematic review and plasma cholesterol predictive equations estimations
Source: Nutr Rev. 2023 Sep 11;82(8):1079–86. doi: 10.1093/nutrit/nuad109 (PMC11233854; doi:10.1093/nutrit/nuad109)
Supplement: nuad109_Supplementary_Data [file nuad109_supplementary_data.docx]

Table S1. Qualitative summary of studies identified by the systematic search strategy and excluded from full-text review

| **Study** | **Design** | **Duration (days)** | **Participant Characteristics** | **Sample Size** | **Intervention** | **Control** | **Outcome Measure** | **Results** | **Reason for Exclusion** |
| --- | --- | --- | --- | --- | --- | --- | --- | --- | --- |
| Anderson et al., 1957^1^; experiments C, F, H | Nonrandomized parallel controlled study | 120 | Adults, 32-62y, diagnosis of schizophrenia | n = 9-26 per experiment; 100% M | C: isocaloric substitution of a 50 gm daily of OO or  CSO for CHO in a low-fat or butterfat diet  F: isocaloric substitution of a 100 gm daily of OO or  of CSO for CHO in a low-fat or butterfat diet  H: isocaloric substitution of a 100 gm daily of corn oil, hydrogenated coconut oil or CSO for CHO in a low-fat diet | Low fat Diet without CHO substituted or House Diet | Serum cholesterol | C: No significant difference between the effects of OO and CSO. Low-fat diet was more effective than experimental diets in lowering the serum cholesterol concentration from the House Diet.  F: Low-fat and CSO decreased serum cholesterol compared to butterfat and House Diet  H: Low-fat and CSO decreased serum cholesterol compared to butterfat and House Diet, but corn oil produced the greatest decrease | Study design |
| Higazi et al., 1971^2^ | Nonrandomized parallel controlled study | 8 | Adults, 28-75y; healthy or atherosclerotic | n=56, M & F | CSO, Corn oil, egg yolk, cow butter effects in people with atherosclerosis | CSO, Corn oil, egg yolk, cow butter effect is people who are healthy | Effect of acute fat loads on serum TG and chylomicrons | Plant fats led to less increase in TG and chylomicrons. Corn oil increased values less than CSO | Study design |
| Matthew and Dean, 1960^3^ | Nonrandomized parallel controlled study | 21 | Children with Kwashiorkor | n=142, 10-36mo, M & F | 1: Protein same as control, butter added 2: Protein same as control, CSO added 3: 1/8 of the protein from dried skimmed milk, 3/4 from groundnuts, and 1/8 from a cereal mixture; fat from the groundnuts and CSO | Fat free diet: Protein from calcium caseinate and dried skimmed milk | Serum cholesterol | CSO increase in TC (135 mg/100 mL serum) was less than butter and more than biscuit at 7 days. CSO increase in TC was lower than butter at 14 and 21 days. | Study design |
| Wilcox and Gallaway, 1961^4^ | Nonrandomized crossover controlled study | 15 | Young adults, healthy | n=16; n=8 per group, 50% M | 35% butter, margarine, partially hydrogenated CSO, CSO, lard, partially hydrogenated lard, corn oil | 35% CSO, lard or butter | Serum cholesterol | 12% decrease of TC with margarine, partially hydrogenated CSO, and CSO compared with a 22% decrease with corn oil | Study design |
| Mendez et al., 1961 (II. Effect of gross dietary changes)^5^ | Nonrandomized 1: Crossover controlled study  2-4: Parallel controlled study | 1: 28 days 2: 56 days 3: 28 days 4: 28 days | Children in orphanages in Guatemala City | 1: n=26; 85% M  2: n=18; 56% M  3: n=12; 58% M  4: n=12; 67% M | 1: Diets with CSO, hydrogenated CSO or lard as primary fat  2: Mixed fats to simulate those used by high income 3: High in calories from CHO and fat to simulate lower income families 4: Calories from fat simulated upper income families | N/A | Serum cholesterol | 1: CSO increased TC levels from 119 mg to 133 mg, hydrogenated CSO to 147 mg, and lard to 152 mg 2: TC increased from 155 mg/dL to 129 mg/dL 3 & 4: No significant changes | Study design |
| Brown, 1961^6^ | Nonrandomized parallel external controlled study | 18 | Adults, "normal," active | N=40; n=4-6 M & F per experiment | 1:Vegetarian diet with 34% of calories as CSO and 4% basic fat 2: 4% of calories from meat fat, 32% of calories from CSO , 4% basic fat 3: 4% of calories from meat, 1.5% from butter, 1.5% from eggs, 30% CSO, and 3% basic fat | N/A | Serum cholesterol | All diets reduced serum cholesterol 62-65 mg/ 100 mg | Study design |
| Brown et al., 1966^7^ | Nonrandomized parallel controlled study | 18 | Adults, 20-55y, healthy and normocholesterolemic | n=112; n=5-8 per experiment; 86% M | 1: animal fat increased to above the standard diet; total fat was maintained by decreasing vegetable oil 2: same as 1 expect eggs provided additional animal fat  3: type of vegetable oil changed, using hydrogenated products and an unsaturated oil 4: amount of vegetable oil was less than the standard diet  5: diets representing special practical dietary problems | Standard Diet: Vegetable-oil diet with 4% of calories from meat | Serum cholesterol | When CSO was decreased to 55g, serum cholesterol decreased only 9.5% (less than with the standard diet). At 78g CSO TC decreased 23.4%, which is as much as the standard diet. | Study design |
| Gordon et al., 1957^8^ | Nonrandomized uncontrolled study | 12-34 days | Adults, variety of accidents and diseases | n=10, 100% M n=1-3 per experiment; | Varying doses of crude and refined sunflower seed oil, CSO, and hydrogenated coconut fat in isocaloric substitution for sugar and syrup or as supplements | Basal low-fat diet | Serum cholesterol | Sunflower-seed oil lowered the TC level; transitioning from sunflower oil to CSO lead to an increase in TC (participant 2) | Study design |
| Mendez et al., 1961^9^ | Nonrandomized parallel controlled study | 126-315 days | Children in two rural villages in Guatemala | n=101; n=46, n=60 | Group A: 30 g of fat (2 fried tortillas with fried black bean paste with lard) Group B: 30 g of fat (2 fried tortillas with fried black bean paste with hydrogenated CSO) Group C: Plain CSO | N/A | Serum cholesterol | No significant effects of added oils were seen in any group | Study design |
| Davis et al., 2012^10^ | Nonrandomized uncontrolled study | 5 | Adults, 18-40y; healthy | n=38, 32% M | 95 g CSO per day for in one week | N/A | Lipids/  lipoproteins | Participants had a decrease in TC (-0.26 mmol/L) and LDL-C (-0.23 mmol/L) with no change in HDL-C | Study design |
| Shaper et al., 2012^11^ | Nonrandomized cross-sectional study | 1 day | Adults and children from Africa and Asia | n=671; 100% M | N/A | N/A | Mean serum cholesterol levels in different age groups | 12 yr olds from Africa had higher TC (166 mg/dL) than 40 yr olds (145 mg/dL)  12 yr olds from Asia had lower TC (206 mg/dL) than 40 yr old (248 mg/dL) | Study design |
| Ahrens et al., 1957^12^ | Nonrandomized 1: Crossover controlled study  2: Parallel controlled study | 120-180 days | Adults, hypercholesterolemic and hyperlipidemic or normocholesterolemic with arteriosclerotic heart disease | 1: n=8  2: n=32; 65 % M; | Liquid formula with protein 15%, fat 40%, and CHO 45% of total calories with varying amounts of corn oil, lard, CSO, OO, beef tallow etc. | N/A | Serum-lipid levels | The lowest serum-lipid levels were seen when corn oil, safflower-seed oil, or CSO constituted the sole dietary fats | Study design |
| Nestel et al., 1973^13^ | Nonrandomized crossover controlled study | 80 days | Adults with severe obesity, 26-59 y, normocholesterolemic | n=8, 25% M | Two-pool compartmental analysis | Chemical sterol balance techniques | Cholesterol turnover measured by isotopic kinetic analysis versus chemical sterol balance technique | Cholesterol turnover was 10% higher by compartmental analysis | Study design |
| Kinsell et al., 1953^14^ | Nonrandomized uncontrolled study | 4-90 days | Patients on tube feeds | n=~6; n=1 | Various amounts of CSO as fat source | Various amounts of mixed soy and CSO in tube feed | Serum cholesterol | Formula diets containing large amounts of vegetable fat reduced serum cholesterol and phospholipids | Study design |
| Deuel et al., 1949^15^ | Nonrandomized crossover controlled study | 9 days | Chronically ill patients | n=8 | 88% of total fat from CSO | 88% of total fat from rapeseed oil | Digestibility of oils | Average digestibility of rapeseed oil was 99% and 96.5% for CSO | Study design |
| Keys et al., 1957^16^ | Nonrandomized crossover trial | 14-63 days/trial | Adults | n=27; n=12-27/trial, 100% M | Butter, CSO, hydrogenated coconut oil, corn oil, OO, margarine, safflower, sardine oil, sunflower-seed oil or sunflower oil composing 75% of the 9-44% fat of diet | Estimated serum total cholesterol changes | Efficacy of predictive equation for serum total cholesterol predicted changes | Regression equation was valid for predicting cholesterol changes in saturated and polytheoid fats except for corn oil and hydrogenated coconut oil | Study design |
| Knoebel and Nasset, 1957^17^ | Nonrandomized parallel controlled trial | 1 day | Adults | n=6 | Fed 150 g of ice cream with 15 g butterfat | Dogs fed 25 g CSO | Gastric and intestinal hydrolysis of fat | As the percentage of monoglycerides and free fatty acids increase, percentages of diglycerides and triglycerides decrease in the duodenum; similar in human and dog | Study design |
| Karvinen et al., 1957^18^ | Nonrandomized crossover controlled study | 7 days/series | Adults, 21-38y; healthy | n=16, 81% M | 1, 3, 6 or 9 gm of cholesterol added to the daily diet mixed into 17 gm of oleomargarine (hydrogenated soya and CSO) (76.5% fat) | Basal diet of American foods | Cholesterol absorption | Maximum capacity of 2.9 gm of cholesterol absorption | Study design |
| Sivaramakrishnan et al., 1994^19^ | Basic study | 28 days | Mice | N = 30 | Ice cream mix; Ice cream mix with substitution of 20% milkfat with CSO; Ice cream mix with substitution of 15% milk fat with CSO; Ice cream mix with substitution of 15% milk fat with blend of 10% CSO and 5% soyabean oil | Control basal diet | Serum cholesterol | TC of mice is decreasing significantly (p<0.01) as percent replacement of milk fat with CSO increased; decrease of (~2-7 mg/dL) | Population |
| Pinter et al., 1966^20^ | Randomized crossover controlled study | 8 hours | Adults, 23-29y; ambulatory | N=17, 100% M | 50 g fat as hydrogenated CSO or hydrogenated corn oil or 25-50g glyceral monolineate | 50 g fat CSO or corn oil or 25-50g glycerol monostearate | Postprandial serum TG | Unsaturated fats exhibited a rise in serum TG, but not saturated (hydrogenated form). CSO serum TG increased hours 4-6, but no increase in hydrogenated CSO. Both corn oil forms performed similarly | Comparison |
|  |  |  |  |  |  |  |  |  |  |
|  |  |  |  |  |  |  |  |  |  |
| Polley et al., 2018^21^ | Randomized crossover controlled study | 5 | Adults, 18-45y, healthy | N=15, 100% M | 44% of total energy as CSO | 44% of total energy as OO | Metabolism (RER and diet induced thermogenesis) | Higher RER for the CSO pre-diet compared with the OO pre-diet visit (postprandial avg= 0.86) Lower fat oxidation for the CSO pre-diet vs OO pre-diet visits (postprandial avg = 1.200 Higher CHO oxidation for the CSO pre-diet vs OO pre-diet visit (post-prandial avg = 3·30)  Lower diet induced thermogenesis for the CSO pre-diet visit vs OO pre-diet (postprandial avg = 18·91) Greater decrease in CHO oxidation from pre- to post-CSO diet intervention vs OO (postprandial avg change of −0·38) | Outcome |
|  |  |  |  |  |  |  |  |  |  |
| Pinter et al., 1966^22^ | Conference abstract | N/A | N/A | N/A | N/A | N/A | N/A | N/A | Publication type |
| Prater et al., 2021^23^ | Conference abstract | N/A | N/A | N/A | N/A | N/A | N/A | N/A | Publication type |

CHO: carbohydrate; CSO: cottonseed oil; OO: olive oil; MUFA: monounsaturated fatty acids; PUFA: polyunsaturated fatty acids; TC: total cholesterol; TG: triglycerides; RER: respiratory exchange rate

Table S2. Search terms by search engine with filters in parentheses

| PubMed (limit to English) | ("Cottonseed Oil"[MeSH Terms] OR "cottonseed oil" [Text Word] OR "cottonseed oils" [Text Word]) OR (("cottonseed"[Text Word] OR "cottonseeds"[Text Word]) AND ("oil"[Text Word] OR "oils"[Text Word])) NOT (“animals”[mesh] NOT (“humans”[mesh] AND “animals”[mesh])) |
| --- | --- |
| Clinicaltrial.gov | Cottonseed OR Cottonseeds |
| Cochrane CENTRAL | Cottonseed* |
| CINAHL (limit to English) | (MH "Cottonseed Oil") OR cottonseed* |
| CAB | (("cottonseed oil" OR "cottonseed oils")) AND ( ((organism-descriptor:(( "man" ) )) (language:(( "English" ) )) )) |

References

1. Anderson JT, Keys A, Grande F. The effects of different food fats on serum cholesterol concentration in man. *J Nutr.* 1957;62:421-444.

2. Higazi AM, El-Ebrashy N, El-Ahmady L, El-Ashmawy S, Khalafallah A, Gaber A. Changes in serum triglycerides and blood coagulation in response to acute fat loads in atherosclerotics. *Journal of the Egyptian Medical Association.* 1971;54(10):659-670.

3. Matthew CE, Dean RFA. The serum lipids in kwashiorkor. 2. The relation of diet to total serum cholesterol. *Journal of Tropical Pediatrics.* 1960;5:135-140.

4. Wilcox EB, Galloway LS. Serum cholesterol and different dietary fats. *Journal of the American Dietetic Association.* 1961;38(3):227-230.

5. Mendez J, Scrimshaw NS, Flores M, De Leon R, Behar M. Factors influencing serum cholesterol levels of Central American children. 2. The effect of gross dietary changes. *American Journal of Clinical Nutrition.* 1961;9:148-153.

6. Brown HB. Fashioning a practical vegetable-oil food pattern. An experimental study. *Journal of the American Dietetic Association.* 1961;38:536-539.

7. Brown HB, Farrand M, Page IH. Design of practical fat-controlled diets. Foods, fat composition, and serum cholesterol content. *Journal of the American Medical Association.* 1966;196:205-213.

8. Gordon H, Lewis B, Eales L, Brock JF. Dietary fat and cholesterol metabolism. Faecal elimination of bile acids and other lipids. *Lancet.* 1957;2:1299-1306.

9. Mendez J, Scrimshaw NS, Ascoli W, Guzman MA. Factors influencing serum cholesterol levels of Central American children. I. Effect of adding fat and animal protein to the diet. *American Journal of Clinical Nutrition.* 1961;9(2):143-147.

10. Davis KE, Prasad C, Imrhan V. Consumption of a diet rich in cottonseed oil (CSO) lowers total and LDL cholesterol in normo-cholesterolemic subjects. *Nutrients.* 2012;4(7):602-610.

11. Shaper AG, Jones KW. Serum-cholesterol, diet, and coronary heart-disease in Africans and Asians in Uganda. *Lancet.* 1959;2:534-537.

12. Ahrens EH, Jr., Hirsch J, Insull W, Jr., Tsaltas TT, Blomstrand R, Peterson ML. The influence of dietary fats on serum-lipid levels in man. *Lancet.* 1957;272:943-953.

13. Nestel PJ, Schreibman PH, Ahrens EH, Jr. Cholesterol metabolism in human obesity. *Journal of Clinical Investigation.* 1973;52(10):2389-2397.

14. Kinsell LW, Michaels GD, Partridge JW, et al. Effect upon serum cholesterol and phospholipids of diets containing large amounts of vegetable fat. *Am J Clin Nutr.* 1953;1:224-231.

15. Deuel HJ, Jr., Greenberg SM, Anisfeld L, Melnick D. The effect of fat level of the diet on general nutrition. VIII. The essential fatty acid content of margarines, shortenings, butters, and cottonseed oil as determined by a new biological assay method. *J Nutr.* 1951;45(4):535-549.

16. Keys A, Anderson JT, Grande F. Prediction of serum-cholesterol responses of man to changes in fats in the diet. *Lancet.* 1957;273:959-966.

17. Knoebel LK, Nasset ES. The digestion and absorption of fat in dog and man. *Journal of Nutrition.* 1957;61:405-419.

18. Karvinen E, Lin TM, Ivy AC. Capacity of human intestine to absorb exogenous cholesterol. *Journal of Applied Physiology.* 1957;11:143-147.

19. Sivaramakrishnan R, Narasimhan R, Thangavel K, Khan MMH. Studies on partial substitution of milk fat with vegetable oils in softy ice cream. *Cheiron.* 1994;23(1):9-14.

20. Pinter KG, Karle IP. Effect of ingestion of various mono- and triglycerides on serum triglyceride concentration. *Am J Clin Nutr.* 1966;18(3):165-168.

21. Polley KR, Miller MK, Johnson M, Vaughan R, Paton CM, Cooper JA. Metabolic responses to high-fat diets rich in MUFA v. PUFA. *Br J Nutr.* 2018;120(1):13-22.

22. Pinter KG, Goldsmith GA, Itallie TBV. Effect of various fats on cholesterol ester metabolism in man. *American J of Clin Nutr.* 1966;18(4):305.

23. Prater MC, Scheurell A, Paton C, Cooper J. Comparison of appetite responses from diets enriched with cottonseed oil versus olive oil. *Obesity (Silver Spring, Md).* 2021;29(SUPPL 2):75‐76.
